# Detection of Cellular Senescence in Human Primary Melanocytes and Malignant Melanoma Cells In Vitro

**DOI:** 10.3390/cells11091489

**Published:** 2022-04-28

**Authors:** Tom Zimmermann, Michaela Pommer, Viola Kluge, Chafia Chiheb, Susanne Muehlich, Anja-Katrin Bosserhoff

**Affiliations:** 1 Institute of Biochemistry, Friedrich-Alexander-Universität Erlangen-Nürnberg (FAU), 91052 Erlangen, Germany; tom.zimmermann@fau.de (T.Z.); michaela.pommer@fau.de (M.P.); viola.kluge@fau.de (V.K.); chafia.chiheb@fau.de (C.C.); 2 Department of Chemistry and Pharmacy, Friedrich-Alexander-Universität Erlangen-Nürnberg (FAU), 91052 Erlangen, Germany; susanne.muehlich@fau.de; 3 Comprehensive Cancer Center (CCC) Erlangen-EMN, 91054 Erlangen, Germany

**Keywords:** senescence, melanocyte, melanoma, beta-galactosidase

## Abstract

Detection and quantification of senescent cells remain difficult due to variable phenotypes and the absence of highly specific and reliable biomarkers. It is therefore widely accepted to use a combination of multiple markers and cellular characteristics to define senescent cells in vitro. The exact choice of these markers is a subject of ongoing discussion and usually depends on objective reasons such as cell type and treatment conditions, as well as subjective considerations including feasibility and personal experience. This study aims to provide a comprehensive comparison of biomarkers and cellular characteristics used to detect senescence in melanocytic systems. Each marker was assessed in primary human melanocytes that overexpress mutant BRAFV600E, as it is commonly found in melanocytic nevi, and melanoma cells after treatment with the chemotherapeutic agent etoposide. The combined use of these two experimental settings is thought to allow profound conclusions on the choice of senescence biomarkers when working with melanocytic systems. Further, this study supports the development of standardized senescence detection and quantification by providing a comparative analysis that might also be helpful for other cell types and experimental conditions.

## 1. Introduction

Cellular senescence describes a stable state of growth arrest, commonly accompanied by ample molecular and phenotypical changes. In the decades since its first description by Hayflick and Moorhead in 1961 [1], cellular senescence has been linked to numerous physiological and pathological conditions, ranging from developmental processes to neurodegenerative diseases and cancer [2]. In each of these conditions, establishment of senescence is caused by one of three major mechanisms: telomere shortening, oncogene activation, or extensive DNA damage [3]. When it comes to melanocytes and malignant melanoma, two of these mechanisms are of special importance: first, oncogene-induced senescence (OIS) as a central feature of melanocytic nevi that prevents further oncogenesis and malignant transformation of such benign lesions [4]. As melanocytic nevi have been causally linked to development of malignant melanoma [5,6], stabilization of OIS or clearing of senescent melanocytes remain an important and promising approach in preventing tumorigenesis [7,8]. Second, DNA damage-induced senescence is an important part of therapeutic treatment of malignant melanoma. The majority of cytotoxic treatments cause DNA damage to induce apoptosis and senescence, thereby halting tumor growth [9]. However, growing evidence was found that senescent cancer cells might become therapy-resistant, resulting in residual tumor masses and potentially recurrent malignancies [10,11]. Consequently, there is a need for efficient and reliable detection and targeting of senescent cells. Although fundamental hallmarks of cellular senescence are conserved in most experimental and clinical conditions, the exact phenotype is often variable and affects the reliability of biomarkers [12]. In addition, the majority of these markers are not completely specific for cellular senescence, e.g., growth arrest and activation of DNA damage response [12,13]. Melanocytes introduce another potential problem since they physiologically show high levels of lysosomal beta-galactosidase activity [14]. This potentially interferes with detection of senescence-associated beta-galactosidase (SA-β-Gal) [15], one of the most widely used markers of cellular senescence [16], and needs to be taken into consideration. The aim of this study is to comprehensively evaluate biomarkers of cellular senescence for their use in primary melanocytes and melanoma cells. It is thought to support the ongoing discussion on the choice of the best markers, especially in the complex field of melanoma research, and thereby improving the reliability and reproducibility of senescence detection.

## 2. Materials and Methods

### 2.1. Cell Culture

Normal human melanocytes (NHEM, neonatal) were obtained from Lonza and cultivated in MGM-4 BulletKit medium (Lonza, Basel, Switzerland) with 1% penicillin/streptomycin. NHEM from different donors were used between passages 6 and 8. Cells from the same donor, but at a different passage, were considered biological replicates. HEK293T cells for transduction were a generous gift from Prof. Stephan Hahn (Ruhr-Universität Bochum, Germany). Their cultivation required high-glucose Dulbecco’s modified Eagle’s medium (DMEM) with 10% FCS and 1% penicillin/streptomycin. Both NHEM and HEK293T cells were incubated at 37 °C and 5% CO_2_ in a humified atmosphere. Melanoma cell line Mel Juso was cultivated in RPMI 1640 medium with 2% sodium bicarbonate, 10% FCS and 1% penicillin/streptomycin at 37 °C, and 8% CO_2_. Mycoplasma contamination was regularly excluded for all primary cells and cell lines. When reaching approximately 80% confluence, cells were washed with PBS and detached using a solution of 0.05% trypsin and 0.02% EDTA in PBS. After centrifugation and removal of the trypsin solution, cells were either passaged or counted using a Neubauer counting chamber. Melanoma cell line Mel Im was cultured as described in Section 2.3. Unless otherwise stated, cell culture chemicals and media were obtained from Sigma Aldrich (Steinheim, Germany).

### 2.2. Lentiviral Transduction of Melanocytes

Lentiviral transduction using a third-generation vector system was described elsewhere [17]. Briefly, HEK293T cells were seeded in 10 cm plates at a density of 2 × 10^6^ cells/plate. On the next day, three vectors were introduced simultaneously using transfection with Lipofectamine^®^ LTX (Thermo Fisher, Waltham, MA, USA): an envelope plasmid pHIT-G, a packaging plasmid pCMV ΔR8.2, and a target plasmid with the DNA of interest (either copGFP or B-RAF^V600E^). Cells were incubated 16 h before the medium was changed to MGM-4 BulletKit medium. After additional incubation for 24 h, supernatants were collected, filtered, and applied to NHEM. Polybrene^®^ (Santa Cruz, Dallas, TX, USA) was added to a final concentration of 1 µg/mL to increase the efficiency of viral uptake. Due to high sensitivity of primary cells, lentiviral supernatants were removed after approximately 6 h. Cells were washed three times with PBS and cultivated in regular MGM-4 BulletKit medium. All experiments using transduced melanocytes started exactly 7 days after transduction to allow establishment of a senescent phenotype. All data in this study are derived from samples that are either untransfected or transfected with scrambled siRNAs. Different transductions are referred to as Mock (copGFP control plasmid) or BRAFm (B-RAFV600E).

### 2.3. Induction of Senescence in Melanoma Cells

Etoposide treatment started 24 h after approximately 200,000 cells/well were seeded in 6-well plates. Etoposide (R&D Systems, Minneapolis, MN, USA) was dissolved in DMSO to achieve a stock solution of 50 mM, which was then diluted in culture medium to a final concentration of 100 µM and applied to the cells. Control cells were treated with a similar amount of DMSO to exclude effects of the solvent. After an incubation period of 48 h, cells were detached as described in Section 2.1 and either collected for further processing or counted using a Neubauer counting chamber.

Treatment with acidified nitrite was described recently [18]. After a treatment period of 5 min, cells were incubated for 48 h in culture medium and eventually detached and collected for further processing. For the analysis of long-time acidosis effects, melanoma metastasis cell line Mel Im was cultured in medium at pH 6.7 for at least 2 months prior to analyzation. Therefore, low-glucose DMEM was supplemented with 10% FCS and 1% penicillin/streptomycin and 0.2% sodium bicarbonate as buffer. Cells were then incubated at 37 °C and 8% CO_2_ to set the desired pH value. Control cells were cultured conventionally at pH 7.4 in low-glucose DMEM including 3.7% sodium bicarbonate, supplemented with 10% FCS and 1% penicillin/streptomycin at 37 °C, and 8% CO_2_.

### 2.4. Analysis of mRNA Expression Using Real-Time PCR

Total RNA isolation was achieved using E.Z.N.A.^®^ Total RNA Kit II (Omega Bio-Tek, Norcross, GA, USA) according to manufacturer’s instructions. Generation of cDNA was performed as previously described [19]. For real-time PCR, LightCycler^®^ 480 II devices (Roche, Basel, Switzerland) were used with forward and reverse primers from Sigma-Aldrich. Primer sequences can be found in Table 1.

### 2.5. Western Blot Protein Analysis

Total protein isolation was realized using radio-immunoprecipitation assay buffer (Roche) as described previously [17]. A total of 20 µg protein were loaded on 12.75% SDS polyacrylamide gels for electrophoresis and immediately blotted onto a PVDF membrane (Bio-Rad, Hercules, CA, USA). Protein load of each sample was quantified using Ponceau S staining. After a washing step using double distilled water, membranes were blocked for 1 h using 5% non-fat dried milk/TBS-T. Primary antibodies against γH2AX (1:1000 in 5% NFDM, Cell Signaling, 9718), p16 (1:500 in TBS-T, R&D Systems, AF5779, previously used in [20]), p21 (1:1000 in 5% NFDM, Abcam, ab109199), p53 (1:2000 in 5% BSA, Santa Cruz, sc-126), pERK and ERK (1:1000 in 5% BSA, Cell signaling, 4370 and 9102) were incubated overnight, shaking at 4 °C. The primary antibody against β-actin (1:5000 in 5% BSA, Sigma Aldrich, A5441) was incubated for 1 h at room temperature. Secondary antibodies conjugated to horseradish peroxidase (HRP, Cell Signaling 7074 and 7076, and Dako P0449) were applied for 1 h at room temperature. Afterwards, Clarity™ Western ECL Substrate (Bio-Rad) was added to the membranes for visualization with a Chemostar chemiluminescence imager (Intas, Goettingen, Germany). Quantification of signal intensity was achieved using LabImage software (Version 4.2.3, Kapelan Bio-Imaging GmbH, Germany). One sample of NHEM was excluded from analysis of p16 protein levels due to artifacts interfering with signal quantification.

### 2.6. Immunofluorescent Stainings

Approximately 20,000 cells were seeded on 18 mm round coverslips and incubated overnight. On the next day, cells were washed twice with PBS and subsequently fixed with 4% PFA for 10 min. Staining procedure started immediately after this fixation step, since even short-term storage was found to interfere with nuclear PML signal in our experiments. Permeabilization using 0.1% Triton-X100 in PBS for 3 min was followed by 30 min blocking with 10% BSA in PBS. The primary antibody against PML (1:200 in 1.5% BSA/PBS, Santa Cruz, sc-966) was added and incubated overnight at 4 °C. The secondary antibody (1:400 in 1.5% BSA/PBS, Thermo Fisher, A32727) was incubated for 1 h at room temperature. Cells were then stained with DAPI (1:10,000 in PBS, Sigma Aldrich) and mounted on microscope slides using Aqua-Poly/Mount (Polysciences, Warrington, PA, USA). Final stainings were analyzed using an Olympus IX83 inverted microscope in combination with Olympus CellSens Dimension software (Version 2.3, Olympus, Tokyo, Japan). DAPI staining of the stainings was used to analyze heterochromatin formation. Brightness and contrast of representative images were adjusted evenly to increase visibility of the staining.

### 2.7. Real-Time Cell Proliferation Analysis (RTCA)

Proliferation was measured using the xCELLigence System (Roche) as described elsewhere [21]. In short, approximately 3000 cells/well were seeded on specific plates and loaded into the device. Proliferation was monitored for five (Mel Juso) or nine days (NHEM) without replacing culture medium. The parameter *slope* describes the steepness of each curve during proliferation and was normalized to control treatment.

### 2.8. XTT Cell Viability Assay

Approximately 3000 cells/well were seeded in a 96-well plate. Cell viability was assessed after an incubation period of 7 days (NHEM) or 48 h (Mel Juso) using the Cell Proliferation Kit II (Roche) according to the manufacturer’s instructions. Due to low cell density, it was not necessary to replace culture medium at any time during the incubation period. A Clariostar Plus Multiplate reader (BMG Labtech, Ortenberg, Germany) was used for photometric detection. Absorbance values were normalized to control treatment.

### 2.9. Staining of Senescence-Associated Beta-Galactosidase Activity

Quantification of β-galactosidase activity was done 7 days post transduction in NHEM and 48 h post treatment in Mel Juso. Fixation and staining were performed using the senescence β-galactosidase staining kit (Cell Signaling, Danvers, MA, USA) according to the manufacturer’s instructions. After staining, cells were washed twice with PBS and stored at 4 °C for up to two weeks. An Olympus IX83 inverted microscope in combination with Olympus CellSens Dimension Software (Olympus) was used to acquire images of the stainings, which were then quantified manually using ImageJ. The same images were used to manually assess and quantify changes in cellular morphology. Brightness and contrast of representative images were adjusted evenly to increase visibility of the staining.

### 2.10. Flow Cytometry of Fluorescent Beta-Galactosidase Substrates

Activity of β-galactosidase was also quantified using fluorescent substrates in combination with flow cytometry. After treatment and adequate incubation times (see Section 2.2 and Section 2.3), approximately 350,000 cells were seeded in 6-well plates exactly 12 h prior staining. The ImaGene Red^™^ C_12_RG lacZ Gene Expression Kit (Molecular Probes) was used in accordance to the manufacturer’s instructions. In short, staining began by addition of 300 µM chloroquine reagent in 1 mL prewarmed cell medium. After incubation for 30 min at 37 °C, 6.67 µL substrate reagent were added directly to the supernatant to achieve a final concentration of 33 µM. Cells were incubated for another 1 h at 37 °C before they were detached and collected. Following centrifugation, samples were resuspended in 1 mM PETG reagent in 1% BSA/PBS and transferred to FACS tubes. For staining with DDAO, a solution of 20 µM DDAO galactoside (Thermo Fisher) and 0.1 µM Bafilomycin A1 (Sigma Aldrich) cell culture medium was prepared and applied to the cells. Plates were then sealed with parafilm and incubated for 90 min at 37 °C. Cells were eventually washed with PBS and detached, followed by centrifugation and resuspension in 1% BSA/PBS. All samples were measured using a BD LSRFortessa™ flow cytometer in combination with BD FACSDiva^™^ software (Version 8.0, BD Biosciences, San Jose, CA, USA).

### 2.11. Statistical Analysis

Analysis and visualization of experimental results was done using GraphPad Prism 9 software (Version 9.1.2, GraphPad Software Inc., San Diego, CA, USA). If not otherwise stated, at least three biological replicated were measured and statistical analysis was performed by Student’s unpaired *t*-test. All results are normalized to the respective control treatment and shown as mean ± SEM. A critical value of *p* < 0.05 was considered statistically significant.

## 3. Results

### 3.1. RNA Markers of Senescence

**Figure 1:**
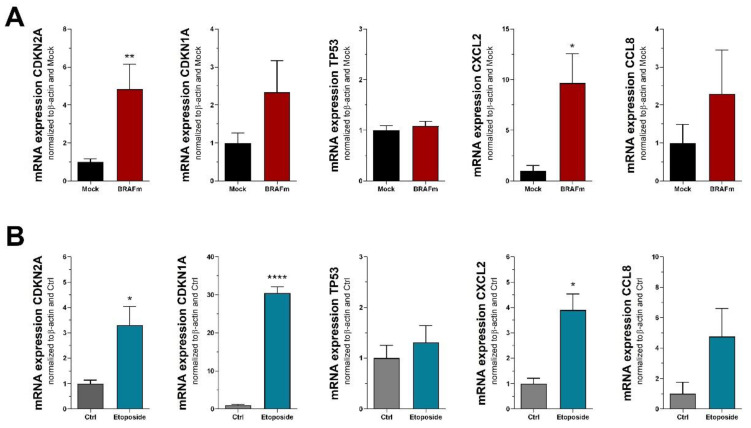
RNA markers of cellular senescence in (**A**) NHEM transduced with mutated BRAF^V600E^ (n = 7) and (**B**) melanoma cell line Mel Juso treated with 100 µM etoposide (n = 3). Bars are shown as mean ± SEM (Student’s *t*-test). (*: *p* < 0.05, **: *p* < 0.01, ****: *p* < 0.0001).

Quantification of gene expression displays an easy and reliable approach to assess cellular conditions, including senescence. We here used quantitative RT-PCR to detect specific mRNAs regulated in senescence of normal human melanocytes (NHEM) and melanoma cells. NHEM received lentiviral transduction of mutated BRAF^V600E^, which leads to oncogene-induced senescence (OIS) as initially described by Michaloglou et al. [4]. Melanoma cell line Mel Juso was treated with 100 µM etoposide, an inhibitor of topoisomerase II, to induce DNA damage and thereby trigger cellular senescence. Traditional mRNA markers of senescence include cell cycle inhibitors and members of the senescence-associated secretory phenotype (SASP). We started with assessing cell cycle inhibitors p21^CIP1/WAF1^ and p16^INK4A^, which are encoded by *CDKN1A* and *CDKN2A* genes, respectively. While CDKN2A was significantly induced in both experimental settings, we detected a significant increase of CDKN1A only in melanoma cells treated with etoposide, while there was no significant effect in senescent NHEM (Figure 1A,B). A third cell cycle inhibitor, p53 encoded by the TP53 gene, did not show any regulation on mRNA level in both systems. We further assessed gene expression of CXCL2 and CCL8, which are associated with the SASP [22]. While both markers were increased in senescent NHEM and melanoma cells, a statistical significance could only be detected for CXCL2.

### 3.2. Protein Markers of Senescence

**Figure 2:**
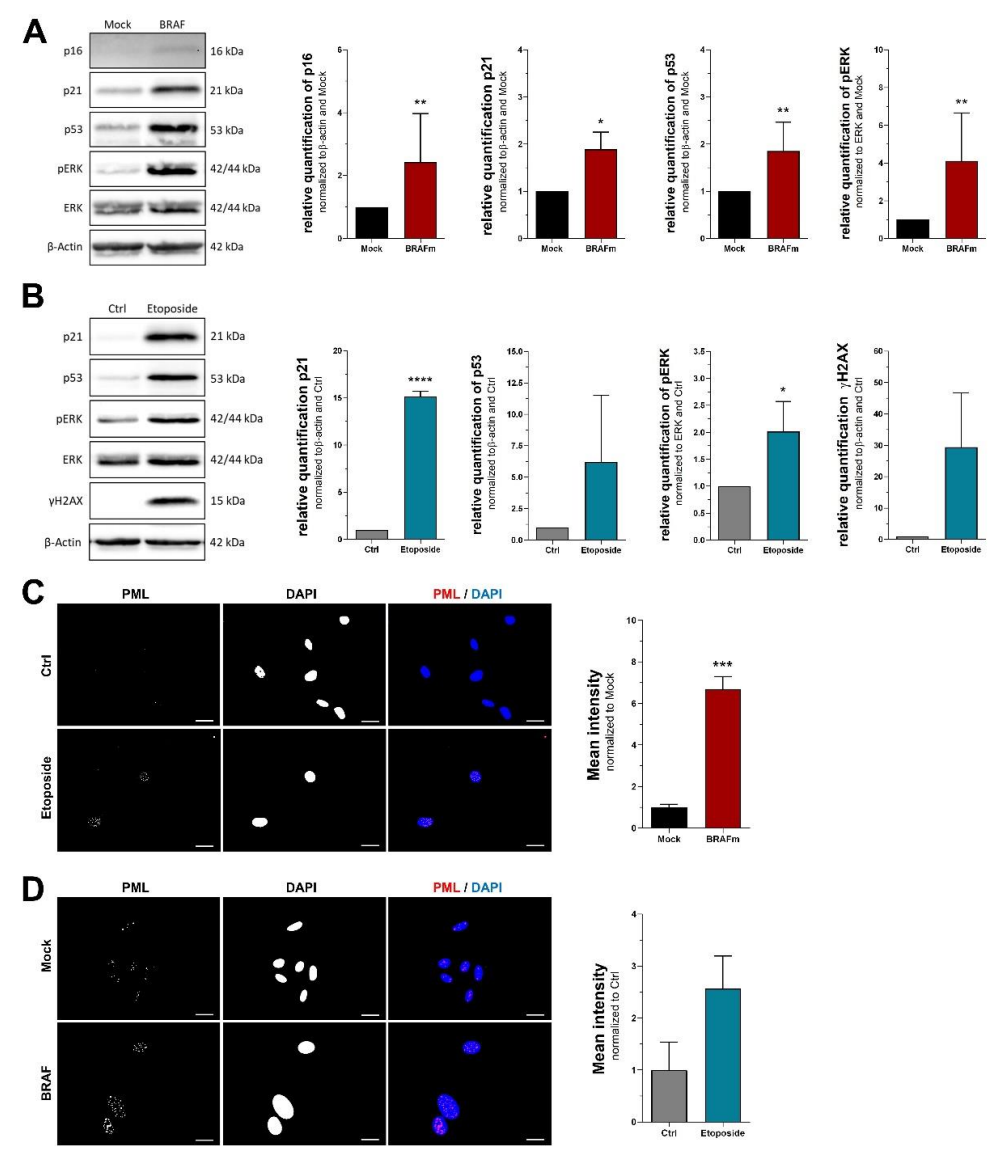
Protein markers of cellular senescence. Western blot analysis of (**A**) NHEM transduced with mutated BRAF^V600E^ (all n = 7, p16 n = 6) and (**B**) melanoma cell line Mel Juso treated with 100 µM etoposide (n = 3). (**C**,**D**) Immunofluorescent stainings of PML and DAPI. Scale bars equal 20 µm. Bars are shown as mean ± SEM (Student’s *t*-test). (*: *p* < 0.05, **: *p* < 0.01, ***: *p* < 0.001, ****: *p* < 0.0001).

Since mRNA markers have a number of limitations, mostly due to the possibility of translational and posttranslational regulation, we next tested for different protein markers of cellular senescence. Interestingly, aforementioned cell cycle inhibitors p16^INK4A^, p21^CIP1/WAF1^ and p53 are among the most common proteins for detection and quantification of cellular senescence [23]. We, therefore, used Western blot analysis to assess and compare their regulation in different experimental settings. Protein levels of all three cell cycle inhibitors doubled during OIS in NHEM (Figure 2A). Melanoma cells treated with etoposide showed a stronger increase of p21^CIP1/WAF1^, while upregulation of p53 turned out to be rather variable (Figure 2B). Cell cycle inhibitor p16^INK4A^ protein, however, was absent in melanoma cells (data not shown). As several studies found an association of ERK1/2 activation with cellular senescence [18,24], we assessed this marker next. A significant increase of phosphorylated ERK1/2 was found in both senescent NHEM and melanoma cells. It is important to note that the effect in NHEM is potentially caused by overexpression of mutant BRAF^V600E^, an upstream kinase of ERK1/2. The melanoma cell line used in our experiments also carries a mutation upstream of ERK1/2, affecting HRAS and NRAS genes. In contrast to NHEM, however, both control and treatment cells bear these mutations, indicating that they do not attribute for the increased phosphorylation of ERK1/2 after etoposide treatment. Furthermore, we detected γH2AX as an indicator of DNA damage in melanoma cells, and found a strong but not reliable upregulation when assessing biological replicates, thereby preventing statistical significance. The same marker could not be detected in senescent NHEM (data not shown). Another molecular marker of DNA damage showed increased levels of nuclear promyelocytic leukemia protein (PML), which is best assessed using immunocytochemistry. While we could detect a significant increase of nuclear PML staining in NHEM, there was only a tendency toward an upregulation in melanoma cells treated with etoposide (Figure 2C,D).

### 3.3. Functional and Morphological Markers of Senescence

**Figure 3:**
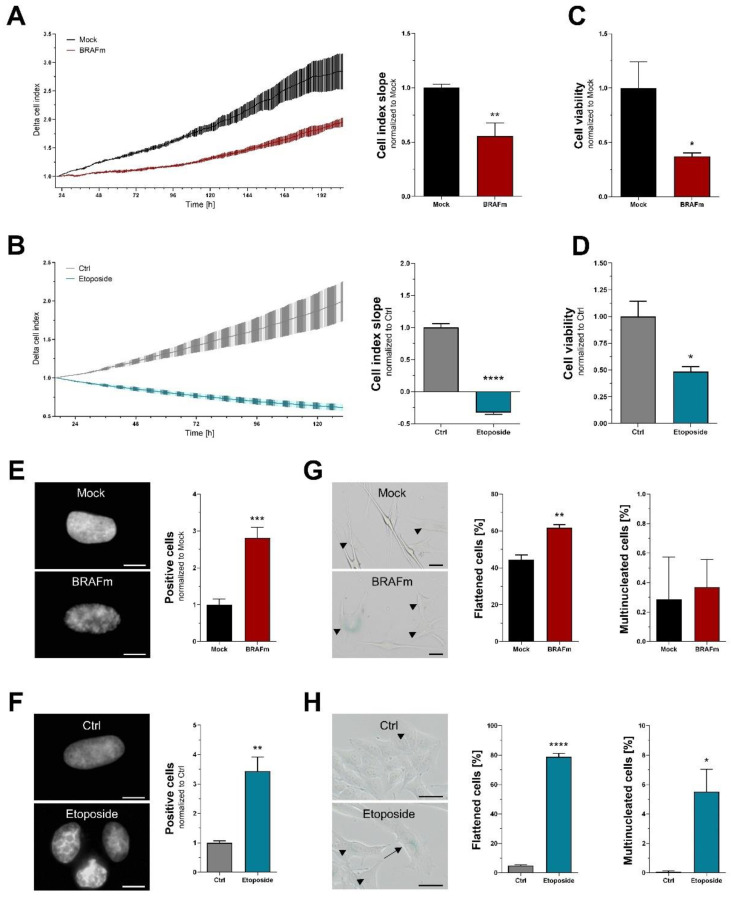
Functional and morphological markers of senescence. RTCA assay using (**A**) NHEM (n = 7) and (**B**) melanoma cell line Mel Juso (n = 3). A representative example is shown on the left. (**C**,**D**) Cell viability analysis 7 days (NHEM, n = 7) or 48 h (Mel Juso, n = 3) after treatment. (**E**,**F**) Detection of cells with visible SAHF using DAPI staining. Scale bars equal 10 µm. (**G**,**H**) Quantification of flattened and multinucleated cells. Arrowheads indicate flattened cells, arrows mark multinucleation. Scale bars equal 20 µm. Bars are shown as mean ± SEM (Student’s *t*-test). (*: *p* < 0.05, **: *p* < 0.01, ***: *p* < 0.001, ****: *p* < 0.0001).

A central hallmark of cellular senescence is the discontinuation of cell division and thereby proliferation. We here used real-time cell proliferation analysis (RTCA) to track cell growth over time, and found a significant decrease in both experimental settings (Figure 3A,B). In addition, proliferative activity can be measured indirectly by incubating cells for an appropriate time period followed by cell viability analysis. Since incubation times are largely dependent on the proliferation rate of control cells, we used 7 days (NHEM) and 48 h (Mel Juso) in our experiments. An XTT assay revealed significantly reduced cell viability in BRAF^V600E^-transduced NHEM and etoposide-treated Mel Juso cells, hence indicating reduced proliferation (Figure 3C,D).

Some functional consequences of cellular senescence lead to morphological changes, including heterochromatin formation, flattening, and multinucleation of cells. Senescence-associated heterochromatin foci (SAHF) were detected using DAPI staining, revealing a significant increase in both primary and melanoma cells (Figure 3E,F). Next, we assessed flattening and multinucleation, which were both significantly elevated after treatment of Mel Juso with etoposide (Figure 3H). NHEM, however, already had high levels of flattened cells in the control treatment, with a small but significant increase after entering OIS (Figure 3G). Interestingly, multinucleation in NHEM was negligible, as only very few cells with two or more nuclei were found.

### 3.4. Quantification of Senescence-Associated Beta-Galactosidase Activity

**Figure 4:**
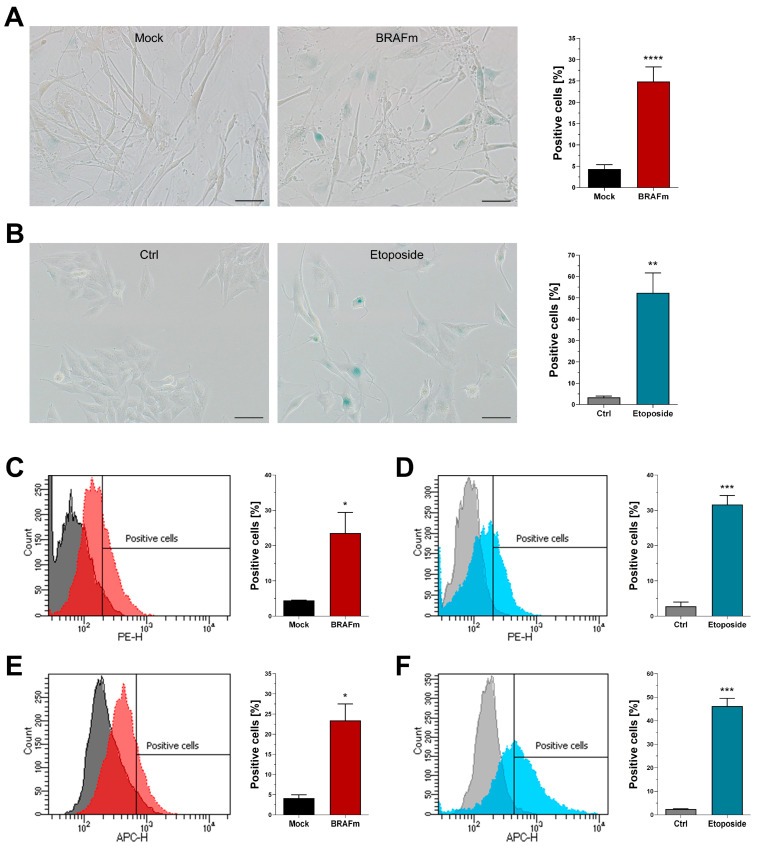
Quantification of SA-β-Gal activity. (**A**,**B**) Traditional method using X-gal and bright field microscopy (NHEM n = 7, Mel Juso n = 3). (**C**,**D**) Flow cytometry after staining with C_12_RG. (**E**,**F**) Flow cytometry after staining with DDAO galactoside. Histograms are representative examples. Scale bars equal 50 µm. Bars are shown as mean ± SEM (Student’s *t*-test). (*: *p* < 0.05, **: *p* < 0.01, ***: *p* < 0.001, ****: *p* < 0.0001).

Next, we quantified activity of senescence-associated beta-galactosidase (SA-β-Gal), which is stated to be the gold standard when it comes to detection of cellular senescence [16]. Multiple experimental approaches have been developed to reliably measure activity of beta-galactosidase in vitro, with the method of Dimri et al. [14] being the most widespread. It is based on the cleavage of X-Gal to yield a blue and insoluble dye, which can be easily detected using bright field microscopy. We used this method to quantify senescent primary and melanoma cells, and detected a significant increase compared to the respective controls (Figure 4A,B). Next, two fluorescent substrates of beta-galactosidase were used in combination with flow cytometry, namely C_12_RG (Figure 4C,D) and DDAO galactoside (Figure 4E,F). While both substrates share the main feature of producing a fluorescent molecule upon hydrolysis by beta-galactosidase, their chemical and functional properties differ notably. The resorufin-based C_12_RG is not fluorescent in its inactive state and carries a lipophilic tail that integrates in the cellular membrane to anchor the fluorescent product within the cell and ensure signal stability. DDAO galactoside, on the other hand, is an intrinsically fluorescent molecule that drastically changes its excitation and emission spectra after hydrolysis. In our experiments, both molecules successfully detected senescent cells similar to the traditional X-Gal assay. The percentage of positively stained cells was comparable among all three detection methods, in both control and treatment settings.

### 3.5. Validating Selected Markers for Detection of Cellular Senescence in Melanoma Cells

**Figure 5:**
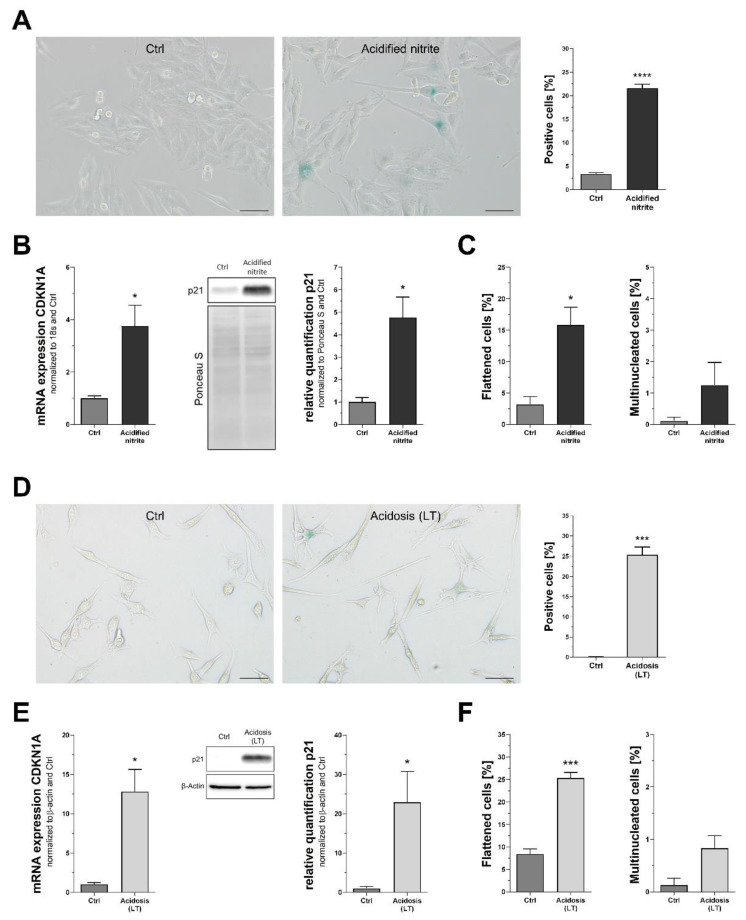
Validation of selected markers in malignant melanoma cells. (**A**–**C**) Mel Juso cells treated with acidified nitrite showed increased SA-β-Gal staining (**A**), p21 mRNA and protein (**B**), and some effects on morphology (**C**). (**D**–**F**) Mel Im cells during long term (LT) acidosis were found to have strong SA-β-Gal activity too (**D**), accompanied by p21 mRNA and protein (**E**) as well as morphological alterations (**F**). Scale bars equal 50 µm. Bars are shown as mean ± SEM (Student’s *t*-test). (*: *p* < 0.05, ***: *p* < 0.001, ****: *p* < 0.0001).

While primary melanocytes are well characterized even in their senescent state, malignant melanoma is one of the most highly mutated cancers and thereby comes with a high grade of heterogeneity [25]. Further, induction of senescence in therapeutic or physiological settings might be due to a broad variety of stimuli, indicating that further validation is necessary for this cell type. Based on the data described in this study, we selected a set of three markers that showed the best results in melanoma cells treated with etoposide: SA-β-Gal, p21, and morphological changes, including flattening and multinucleation. In a first step, Mel Juso cells were treated with acidified nitrite, a novel antitumor treatment previously described [18]. A significant increase of SA-β-Gal, detected via X-Gal assay, was revealed (Figure 5A). Induction of p21 was present on both mRNA and protein level (Figure 5B). Since acidified nitrite interfered with β-actin expression (data not shown), we used 18s mRNA as reference, as well as Ponceau S staining during protein quantification. When assessing morphological changes, we could detect a significant increase of flattened cells, while multinucleation remained scarce (Figure 5C). To account for mutational heterogeneity of malignant melanoma, cell line Mel Im was used in addition. In contrast to Mel Juso cells, which bear a NRAS^Q61L^ mutation but wild-type BRAF, Mel Im cells carry wild-type NRAS but mutated BRAF^V600E^. Further, we introduced a physiological stimulus of cellular senescence, long-term acidosis, as described recently [26]. During long-term acidosis, Mel Im cells exhibited a strong increase of SA-β-Gal staining (Figure 5D), combined with increased p21 mRNA and protein (Figure 5E). Similar to aforementioned treatment with acidified nitrite, we detected elevated levels of flattened cells without any relevant effect on multinucleation (Figure 5F).

## 4. Discussion

Reliable detection and quantification of senescence have been major challenges ever since its first description decades ago. Heterogeneous cell populations, combined with a strong dependency on cell type and senescence trigger [27], increased the difficulty of developing universal molecular markers. To date, the only generally valid marker of cellular senescence is thought to be SA-β-Gal [16], while the vast majority of remaining molecules have to be carefully assessed and validated for each experimental setting. This study focusses on melanocytic systems by comparing two clinically relevant in vitro models: Primary melanocytes bearing mutant BRAF^V600E^ to enter OIS, as commonly found in melanocytic nevi [4], and melanoma cells after treatment with chemotherapeutic agent etoposide. It thereby combines a physiological setting, in which the difference between proliferating and senescent cells is relatively small, with a therapeutic approach that compares highly proliferative cancer cells with severely damaged cells after treatment. The expected distance of control and treatment conditions, in terms of the senescent phenotype, is an important consideration to be made before selection of senescence biomarkers, as it defines the appropriate sensitivity required for the experiment. A second consideration is the quantity of molecular markers. While diagnostic and therapeutic approaches require sophisticated sets of biomarkers to detect senescent cells with great sensitivity and specificity, a reduced and simplified selection might be sufficient for most research applications. The latter will be addressed at the end of this section, as we will propose a condensed set of senescence biomarkers designated for research on melanocytes or melanoma cells.

When assessing cellular senescence, cell cycle inhibitors are commonly used as mRNA and protein markers. Their importance has been extensively reviewed elsewhere [28,29,30]. Briefly, two different pathways are induced, namely Arf/p53/p21 and p16/pRb, both resulting in cell cycle arrest as reviewed by Larsson [31]. However, several limitations have to be taken into account: first, activation of cell cycle inhibitors is dynamic and depends on the cellular state. As indicated by several studies, p21^CIP1^ and p53 are commonly found during the initiation of senescence, but may decline afterwards, while p16^INK4A^ shows delayed upregulation to stabilize senescence [32,33]. Consequently, assessing single cell cycle inhibitors might not be sufficient to detect senescent cells reliably. A second limitation is introduced by their low specificity, especially p21^CIP1^ and p53, as other cellular conditions can cause a similar upregulation. This includes quiescence [34,35], apoptosis [36,37], and cellular dormancy [38]. Finally, cancer cells commonly bear mutations of cell cycle inhibitors [39], potentially interfering with their re-activation and limiting their use as a molecular marker of senescence. As pathways for induction of senescence are far from being fully understood, establishment of a senescent phenotype without activation of major cell cycle inhibitors seems reasonable. This is of special importance for malignant melanoma, as it belongs to the most highly mutated cancers [25].

The SASP is an important hallmark of cellular senescence and can be detected either by qPCR to measure mRNA levels of SASP components or via enzyme-linked immunosorbent assay (ELISA). We here used qPCR to detect levels of CXCL2 and CCL8, while many more SASP components might be equally useful [40,41]. Most of these markers, however are easily affected by cell type, senescence trigger, and cellular microenvironment [42]. Further, SASP components are reportedly increased during quiescence and even apoptosis [43,44]. Moreover, DNA damage is considered to a central mediator of cellular senescence, as it was previously linked to replicative as well as premature senescence caused by oncogene activation, cytotoxic therapy, or other triggers [45]. We assessed two members of the DNA repair response (DRR), gamma-H2AX and PML, which both turned out to be rather unreliable. A possible explanation is found in the experimental settings used in this study: gamma-H2AX is one of the first steps for recruitment and localization of DRR proteins [46], which possibly explains why it could not be detected in primary melanocytes seven days after oncogene overexpression in full senescence. The exact function and regulation of PML during DRR remains unclear, but it was found to be more stable in our experiments. However, it is important to note that PML has widespread cellular functions and is involved in several physiological and pathological processes [47,48]. Another, yet uncommon marker of senescence is phospho-ERK1/2 as an indicator of MAPK activity. Although it seems counterintuitive at first, recent studies have reported significant evidence that MAPK signaling contributes not only to proliferation, but also to cellular senescence [49,50]. Since pathways regulating ERK activation are affected by mutations in many cancers and the majority of melanomas [51], special caution should be exercised when using phospho-ERK1/2 for detection of senescence.

Discontinuation of proliferation displays the most important functional consequence of cellular senescence. A common and feasible method to detect this is metabolic assays based on mitochondrial activity, including XTT assay used in this study. Such indirect measurement of proliferation has certain drawbacks, as mitochondrial activity might be affected without any further consequences on proliferation, leading to false positive results. On the other hand, senescence itself was shown to affect mitochondria [52], thereby impairing the necessary correlation of mitochondrial activity and cell count. Consequently, metabolic assays have only limited reliability when measuring proliferation rates in the context of cellular senescence. Impedance-based systems like RTCA bypass these problems by directly quantifying the coverage of specific cell culture plates, which is a result of cell count and cell size. Although senescent cells commonly show changes in morphology and size [53], such effects can easily be excluded by analyzing the slope of the proliferation curve rather than raw impedance values. Morphological changes might serve as markers of senescence on their own, with the main advantage that they do not require any processing or staining. Senescent cells are usually flattened [53], which was supported by our experiments. Multinucleation could not be found in melanocytes, but melanoma cells treated with etoposide, the reason for which is unknown. Further, there was no relevant increase of multinucleation when testing different senescence inducers and a second melanoma cell line, indicating that this parameter is not reliable. Since research on morphological changes during senescence and underlying mechanisms is sparse, critical evaluation of their use as markers of senescence is barely possible.

Finally, we assessed the gold standard of senescence detection, SA-β-Gal. Its main advantages include easy detection and comparatively high, but not perfect specificity for senescent cells [54]. Melanocytes are among the very few cell types that physiologically express high levels of lysosomal β-galactosidase, the same enzyme referred to as SA-β-Gal in senescent cells [14,15]. Since its expression increases over time, it is generally advisable to use neonatal melanocytes for in vitro experiments, as it was done in this study. Furthermore, experiments including SA-β-Gal should be conducted at low passage numbers. From our experience, primary neonatal melanocytes start to show increased β-galactosidase activity after approximately 15 population doublings (equals 10 passages), which is why all experiments in this study were performed before cells had doubled 12 times (equals 8 passages). We then used two fluorescent substrates in combination with flow cytometry to measure SA-β-Gal and got results comparable to the traditional X-Gal assay, thereby confirming their validity. Flow cytometry has a number of advantages, including the possibility to analyze full samples with a consistent threshold, instead of manually analyzing a small percentage of cells and individually defining positive and negative cells. Pigmented melanocytes introduce another challenge during analysis of X-Gal stainings, as it may be difficult to distinguish between brown pigment and blue dye. The combination of fluorescent substrates and automated analysis using flow cytometry displays an easy solution to overcome such difficulties. Finally, neither staining with fluorescent substrates nor flow cytometry require fixation or preprocessing of cells. This introduces the possibility of flow cytometric sorting of cells with increased SA-β-Gal activity, as it was already done in recent studies [55,56].

After evaluating the molecular markers used in this study, it becomes evident that although the majority of them reliably detects senescence, there is no single molecule or cellular property with sufficient specificity to discriminate between senescence and other, possibly related cellular states. A combination of several markers, each with its own advantages and limitations, might display an adequate solution. As described initially, such a set of markers should be adjusted and validated for each cell type. Based on our data, we suggest two different sets of molecular markers for primary melanocytes and melanoma cells: when working with NHEM, increased SA-β-Gal activity should be the first marker and is preferentially assessed using flow cytometry. Cell cycle inhibitor p16^INK4A^ was found to be strongly and reliably induced, thereby rendering it the second best molecule to detect when investigating cellular senescence. As morphological and functional characteristics were somewhat variable in NHEM, we suggest to add either CXCL2 as a marker of the SASP, or PML immunofluorescence for detection of DNA damage as a third marker. In melanoma cells, SA-β-Gal activity also represents the main marker of cellular senescence, with the advantage that detection via traditional X-Gal assay is sufficient. Beside this, morphological flattening and induction of cell cycle inhibitor p21^CIP1/WAF1^ should be used as additional and reliable markers.

In summary, this study assessed a variety of senescence markers in two different melanocytic systems. We found most of them working reliably, but critical evaluation of their capabilities and drawbacks highlighted the importance of elaborate combined solutions. Finally, we proposed a set of up to three molecular markers for primary melanocytes and malignant melanoma cells to ensure reliable detection of cellular senescence in vitro. Our data supports the ongoing discussion and potentially improves senescence detection, until novel and sophisticated molecular markers are found.

## Figures and Tables

**Table 1:** Oligonucleotides used for real-time PCR.

Gene	Forward Primer	Reverse Primer
CDKN2A	GGAGCAGCATGGAGCCTTCGGC	CCACCAGCGTGTCCAGGAAGC
CDKN1A	CGAGGCACCGAGGCACTCAGAGG	CCTGCCTCCTCCCAACTCATCCC
TP53	AAGTCTAGAGCCACCGTCCA	AGTCTGGCTGCCAATCCA
CXCL2	ATCAATGTGACGGCAGGGAAA	CGAAACCTCTCTGCTCTAACAC
CCL8	CCCAGGTGCAGTGTGACATTA	GGGAGGACCCCACAACACTA
18s	TCTGTGATGCCCTTAGATGTCC	CCATCCAATCGGTAGTAGCG

## Data Availability

Not applicable.
